# Facial Nerve Palsy in Hypertriglyceridemia-Induced Pancreatitis: A Case Report and Literature Review

**DOI:** 10.7759/cureus.46714

**Published:** 2023-10-09

**Authors:** Sondos K Khalil, Sulafa K Khalil, Fakhreddin Al Refai, Zahra B. Yousif, Abdul Majeed Maliyakkal, Omar Adil A Madani, Muzamil Musa

**Affiliations:** 1 Internal Medicine, Hamad Medical Corporation, Doha, QAT; 2 Internal Medicine, University of Gezira, Khartoum, SDN; 3 Internal Medicine, Qatar University, Doha, QAT; 4 Medicine, Hamad Medical Corporation, Doha, QAT; 5 Clinical Medicine, Weill Cornell Medicine, Doha, QAT; 6 Clinical Medicine, QU Health, Qatar University, Doha, QAT

**Keywords:** hypertriglyceridemia-induced pancreatitis, pancreatitis, hypertriglyceridemia, facial nerve, facial palsy

## Abstract

Acute pancreatitis is associated with multiple local or systemic complications in response to systemic inflammation that may eventually result in multi-organ failure. Neurological complications are uncommon in acute pancreatitis. Examples include cerebral hemorrhage, infarction, cerebral fat embolism, Wernicke encephalopathy, and cranial nerve (CN) palsy. Facial nerve palsy is a rare event in the setting of acute pancreatitis, with various theories about its etiology and pathophysiology. We report the case of a 46-year-old female who presented with acute pancreatitis secondary to hypertriglyceridemia. She developed right-sided facial palsy on the third day of admission. Her clinical condition improved with standard conservative medical management of acute pancreatitis. Facial nerve palsy improved after a short course of oral glucocorticoids, supportive measures, and physiotherapy. This case demonstrates a rare occurrence of facial nerve palsy in the setting of acute pancreatitis, although the etiopathology behind this manifestation remains unclear.

## Introduction

Acute pancreatitis is an inflammatory process of the pancreas; this inflammation can initiate a cascade of widespread inflammatory processes affecting multiple organs in the body. The clinical course ranges from a mild illness that can be managed conservatively to a severe and complicated condition associated with multiple organ dysfunction, pancreatic necrosis, and persistent organ failure [1]. The prognosis depends on multiple factors, including the development of organ failure and secondary infection of the pancreas or peripancreatic necrosis [2]. Mortality ranges from 3% in people with moderately edematous pancreatitis to 20% in patients with pancreatic necrosis [3]. Neurological manifestations secondary to pancreatitis are rare; cerebral hemorrhage, infarction, cerebral fat embolism, and Wernicke encephalopathy have been reported in the literature [4-6]. Two cases of acute pancreatitis-associated facial palsy have been reported in the literature [7,8], making ours the third reported case. Another case was reported where the patient developed facial palsy and Adie’s pupils following acute pancreatitis [9]; however, viral infections were not investigated, making it weak evidence. The etiopathogenesis of seventh cranial nerve (CN) palsy in the setting of acute pancreatitis is not fully understood; whether CN palsy is secondary to the disease itself or results as a coincidence from a common etiology is not clear. We are reporting a rare case of hypertriglyceridemia-induced acute pancreatitis associated with seventh CN palsy, and we also discuss potential mechanisms contributing to this rare presentation.

## Case presentation

A 46-year-old female presented to the emergency department complaining of severe epigastric abdominal pain for four days, no radiation or referral, no associated nausea or vomiting, and constipation with the last bowel movement four days prior to admission. She also complained of a new-onset bilateral frontotemporal headache, pulsating in nature with severe intensity, that started on the same day, was not relieved by analgesics, and was not associated with diplopia, blurring of vision, fever, or a history of head trauma. There was no limb weakness or loss of consciousness. Other system reviews were unremarkable.

She is a known case of hypertension, type 2 diabetes, and hyperlipidemia. Her medications are amlodipine, aspirin, insulin glargine, insulin aspart, gliclazide, rosuvastatin, and fenofibrate; however, she has been non-compliant with all her medications.

On examination, she was afebrile (36.6°C), her blood pressure was 159/84 mmHg, her respiratory rate was 18 breaths per minute, her pulse rate was 71 beats per minute, and she maintained saturation in room air. Sclerae are anicteric, with eruptive xanthomas around both eyes. An oral examination showed a clear throat. An abdominal examination showed mild epigastric tenderness and no organomegaly, abdominal masses, or ascites. On neurological examination, she was fully conscious, alert, and oriented, with normal cranial nerves and motor and sensory systems. Her cardiovascular and respiratory system examinations were also normal.

Initial blood tests showed normal blood counts and liver function tests. She had hyperglycemia, elevated lipase, and mild hypokalemia. Renal function tests (RFTs) and lipid panels could not be measured initially due to a lipemic blood sample. A repeated sample showed normal RFTs and markedly elevated triglycerides (TGs) > 11.3 mmol/L (normal: <1.7 mmol). The pregnancy test was negative (Table 1).

**Table 1:** Laboratory test (blood) results HbA1C: hemoglobin A1C, beta-hCG: beta-human chorionic gonadotropin

Test	Result	Laboratory reference
Potassium	3.3 mmol/L	3.5-5.3 mmol/L
Adjusted calcium	2.20 mmol/L	2.20-2.60 mmol/L
Random blood glucose	15.5 mmol/L	3.3-5.5 mmol/L
HbA1C%	9.1%	4.8%-6%
Lipase	279 U/L	8-78 U/L
Triglycerides	>11.3 mmol/L	<1.7mmol/L
Cholesterol	>18 mmol/L	<5.2 mmol/L
Serum beta-hCG	0.8 mIU/mL	0-5 mIU/mL

**Figure 1:**
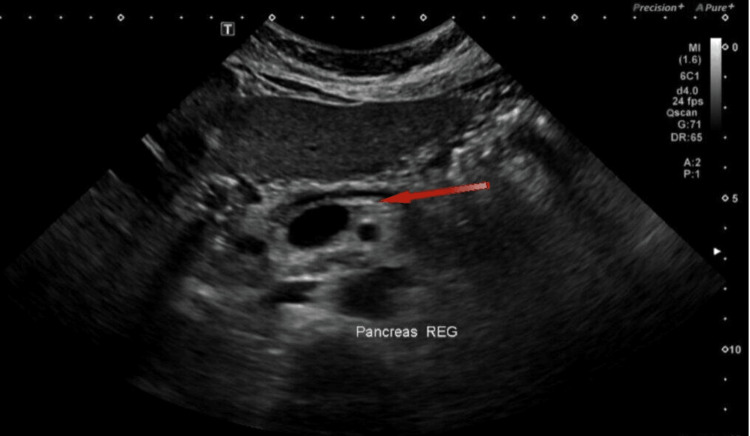
Ultrasound of the abdomen demonstrating normal pancreas echotexture with prominent main pancreatic duct (red arrow)

Ultrasound of the abdomen to look for pancreatitis features was normal (Figure 1). A diagnosis of acute pancreatitis secondary to hypertriglyceridemia was made. She was started on intravenous fluids, insulin intravenous infusion, and rosuvastatin; her antihypertensive medications were resumed to control blood pressure; potassium was replaced; and analgesia pro re nata was given for abdominal pain and headache.

On the second day of admission, her headache improved, but her abdominal pain worsened. A computed tomography (CT) scan of the abdomen was done and was unremarkable. Her triglyceride levels were still unrecordable. Conservative management was continued.

On the third day of admission, her abdominal pain improved, and nausea and vomiting resolved, but she developed right-sided facial droop, with an examination hinting toward lower motor neuron palsy. The rest of the neurological examination was unremarkable. Further workup revealed a negative varicella virus serology. A computed tomography scan of the head did not reveal any significant findings. Her TG level remained elevated, and a diagnosis of right-sided facial palsy was established. She was started on oral prednisolone at 60 mg/day for seven days. She started showing improvement in facial weakness by the sixth day of prednisolone; her abdominal symptoms were completely resolved by that time, with an improvement in TG (5 mmol/L), and she was discharged home.

## Discussion

Facial palsy is a condition in which there is weakness or paralysis of the facial muscles due to seventh cranial nerve palsy. The etiology behind this condition remains unclear; however, Kettel illustrated this in his paper about Bell’s palsy, showing how ischemia due to different reasons may lead to ischemic injury of the nerve, which eventually leads to edema of the nerve and axonotmesis, referring to this process as “dysregulation of the vasa nervorum” [10]. It is worth mentioning that in long-standing cases, the nerve is converted into an atrophic fiber of connective tissue.

While most cases are idiopathic (a condition termed Bell’s palsy, which is a diagnosis of exclusion), there are multiple potential causes of facial palsy, and the first distinction to be made is whether it is due to an upper motor neuron lesion (sparing the forehead) or a lower motor neuron lesion (involvement of the forehead). Important differential diagnoses of the upper motor neuron lesions include subdural hematoma, stroke, neoplasm (e.g., a primary brain malignancy), or multiple sclerosis. Lower motor neuron causes include infection, such as viral infection (including herpes simplex virus 1, cytomegalovirus, and Epstein-Barr virus), acute otitis media, and cholesteatoma; trauma (e.g., temporal bone fracture) or iatrogenic (e.g., mastoid or parotid surgery); and neoplasm (e.g., parotid malignancy) [11].

In our case, the patient presented with facial paralysis after an episode of acute pancreatitis (secondary to hypertriglyceridemia). A local and systemic inflammatory response occurs in the setting of pancreatitis, which promotes a wide range of complications. Facial nerve palsy following acute pancreatitis is a rare event and has been reported only in a few case reports. Bhusal et al. described a 35-year-old male who presented with mild pancreatitis that resolved with treatment. One week following his admission, he presented with right-sided facial palsy, which improved gradually with oral steroids, acyclovir, and physiotherapy [7]. Kim et al. reported a 51-year-old female with no past medical history who presented with right facial nerve palsy while being hospitalized for acute pancreatitis, which was resolved by prednisolone and physical therapy. Two weeks later, she developed a left facial nerve palsy. They concluded that since the interval between the onset of acute pancreatitis and bilateral Bell’s palsy was very close, there was a possibility of a common etiology such as viral infection or autoimmune disease leading to the two conditions [8].

Our patient presented with hypertriglyceridemia, which is another reported factor. As of now, there is no direct, well-established connection between hypertriglyceridemia and facial palsy. However, there are a couple of reported cases of patients presenting with facial palsy in the setting of hypertriglyceridemia. Kudoh and Matsuki reported a 14-year-old male with a history of hypertension and recurrent left facial paralysis due to type 1 hyperlipoproteinemia, which was treated with diet control. Four months later, his laboratory findings showed a marked decline in blood triglycerides; however, his lipoproteins were still high, and other laboratory findings did not show any abnormalities. Facial paralysis was treated with stellate ganglion block and physical therapy. Complete remission of facial paralysis occurred in five months. The authors concluded that the patient’s facial paralysis was probably caused by hyperlipoproteinemia, and hypertension was attributed to hypertriglyceridemia [12].

Alessi et al. suggested in their study that TG, which is associated with atherosclerosis, can contribute to microcirculation disorders due to high levels of fat accumulating in the lining of the blood vessel wall, which promotes endothelial dysfunction and vascular inflammation, causing plaque formation, vascular remodeling, and vascular luminal obstruction, eventually resulting in microvascular ischemia and facial paralysis [13].

Moreover, our patient presenting with high blood glucose (15.5 mmol/L) (normal: 3.3-5.5 mmol/L) and poor glycemic control, evidenced by her high HbA1C% (9.1%), is another associated factor. Many studies have described the relationship between diabetes and facial palsy. Savadi-Oskouei et al. conducted a case-control study that consisted of 403 patients, and they found that diabetes increases the risk for Bell’s palsy [14]. Hung et al. reported that diabetes was significantly higher in the Bell’s palsy group than in the control group in a population-based study in Taiwan [15]. Riga et al. concluded in their cohort study that diabetes with abnormal HbA1C was associated with the severity of Bell’s palsy at initial diagnosis but was not associated with its prognosis, as patients with both normal and abnormal HbA1C showed similar patterns of outcome [16]. Furthermore, diabetes mellitus has been shown to increase the risk of acute pancreatitis. A meta-analysis conducted by Aune et al. illustrated that diabetes mellitus showed a 74% increased risk for acute pancreatitis, a 39% increase in overall pancreatitis risk, and a 40% increase in chronic pancreatitis, which was considered non-specific in the study [17].

## Conclusions

Facial nerve palsy as a complication of acute pancreatitis is rare. This report describes a case of facial palsy that occurred during the course of hypertriglyceridemia-induced acute pancreatitis. Whether nerve palsy is a sequela of the cascade of inflammatory processes associated with acute pancreatitis or a mere coincidence due to other common etiologies remains unclear. We recommend physicians to have a high suspicion of facial palsy as a neurological complication in individuals diagnosed with acute pancreatitis and promptly report it. More studies should be carried out to identify the link between the two conditions to better understand this phenomenon of etiopathogenesis and help identify risk factors and predictive markers, ultimately improving patient outcomes and quality of life.
